# Opportunities for improving data sharing and FAIR data practices to advance global mental health–Corrigendum

**DOI:** 10.1017/gmh.2023.14

**Published:** 2023-04-24

**Authors:** Yaara Sadeh, Anna Denejkina, Eirini Karyotaki, Lonneke I. M. Lenferink, Nancy Kassam-Adams

The authors apologise that upon publication of Sadeh, Y., Denejkina, A., Karyotaki, E., Lenferink, L., & Kassam-Adams, N. (2023). A couple of typographical errors were included.

In Table 2 on the second line the word foster was included incorrectly as ‘DATA Foster PRESERVATION and INDEXING’ this should have said ‘DATA PRESERVATION and INDEXING’

On page 7 the line ‘data reuse of advance science and improve equity’ listed the word ‘of’ instead of ‘to’ and should have read ‘data reuse to advance science and improve equity’
